# Physical activity and exercise beliefs of patients with cancer during chemotherapy: relation with social participation and quality of life level

**DOI:** 10.1007/s00520-026-10893-7

**Published:** 2026-06-20

**Authors:** Ozge Gumus, Ozge Ocag, Sercan Aksoy, Vesile Yildiz-Kabak

**Affiliations:** 1 Institute of Health Sciences, Hacettepe University, Ankara, Türkiye; 2 Fizyoseyhan Wellness Center, Adana, Türkiye; 3 Faculty of Medicine, Department of Medical Oncology, Hacettepe University, Ankara, Türkiye; 4 Faculty of Physical Therapy and Rehabilitation, Hacettepe University, Ankara, Türkiye

**Keywords:** Cancer, Physical activity, Exercise beliefs, Social participation, Quality of life

## Abstract

**Purpose:**

This study aims to examine the physical activity levels and exercise beliefs of patients with cancer receiving outpatient chemotherapy and to investigate their associations with social participation and quality of life.

**Methods:**

A cross-sectional study included 101 outpatient cancer cases undergoing chemotherapy. Physical activity level was assessed by using the International Physical Activity Questionnaire (IPAQ), exercise beliefs with the Exercise Belief Questionnaire. Quality of life with the European Organization for Research and Treatment of Cancer Quality of Life Questionnaire (EORTC QLQ-C30), and social participation with the Reintegration to Normal Living Index (RNLI).

**Results:**

Participants were mostly female (57.4%) and diagnosed with gastrointestinal system (38.6%) or breast cancer (28.7%). According to the IPAQ total score, 44.5% were inactive and 55.4% were minimally active. In terms of exercise beliefs, many participants agreed that exercise makes them feel better (5.4 ± 1.1), positively affects their mood (5.3 ± 1.3), and reduces stress (5.3 ± 1.2). The most common exercise barriers were fatigue (4.5 ± 1.7), bad weather conditions (4.0 ± 2.1), and lack of motivation (3.9 ± 1.9). Patients with higher physical activity levels reported significantly lower exercise barrier (33.4 ± 8.2 vs. 30.0 ± 9.2, *p* = 0.021). Physical activity level was positively correlated with EORTC QLQ-C30 function score (*r* = 0.331, *p* = 0.001), general health score (*r* = 0.247, *p* < 0.05), and RNLI score (*r* = 0.453, *p* < 0.001). Furthermore, positive exercise beliefs were positively correlated with IPAQ total score (*r* = 0.308, *p* < 0.05) and higher perceived exercise barriers were negatively correlated with all EORTC QLQ-C30 quality of life domains (*p* < 0.05), RNLI total score (*r* = −0.388, *p* < 0.001), and IPAQ total score (*r* = −0.273, *p* < 0.05).

**Conclusion:**

Our results suggest that higher physical activity and lower negative exercise beliefs were associated with better social participation and improved quality of life in patients with cancer. To promote better rehabilitation outcomes, future targeted interventions should focus on enhancing patients’ exercise self-efficacy and directly reducing modifiable barriers, such as fatigue and low motivation, alongside reinforcing positive exercise beliefs.

## Introduction

Cancer is a chronic disease that significantly affects individuals’ life and reduces quality of life [1]. Cancer has become increasingly significant in terms of disease burden [2]. If the current trend in cancer continues, 22 million new cases are expected to emerge by 2030 [3]. Improvements in early diagnosis strategies, effective surgical interventions, and medical treatments have led to increased survival rates, making the rehabilitation of survivors even more critical [4]. Cancer patients may experience physical, cognitive, and emotional problems due to disease and cancer treatments. Pain, fatigue, nausea, loss of muscle mass and strength, decreased cardiopulmonary endurance, anxiety, depression, reduced quality of life, and social isolation are only a few of the symptoms caused by treatments. Oncological rehabilitation plays a crucial role in preventing or alleviating these symptoms [5].

Physical activity is one of the most essential components of oncological rehabilitation. Physical activity and exercise have been consistently shown to improve survival outcomes, reduce the incidence of cancer-related side effects, and significantly enhance both the physical and psychosocial well-being of patients during and after treatment [6]. Due to these effects, it has been reported that physical activity and exercise can enhance the quality of life of cancer patients and positively influence their participation in social life [7, 8]. However, another crucial element of cancer rehabilitation outcome, often overlooked, is social participation. Social participation is defined as an individuals’ involvement in life situations and daily roles, encompassing community mobility, leisure activities, and the fulfillment of family roles [9]. Cancer and its intensive treatments, such as chemotherapy, frequently cause physical and emotional side effects that severely disrupt these roles potentially leading to social isolation and reduced engagement in normal living [10].

According to the American College of Sports Medicine, 70% of cancer patients do not comply with physical activity recommendations, and their physical activity levels significantly decrease after diagnosis. Lack of sufficient information and awareness regarding the importance of doing exercise has been cited as a reason for physical inactivity [11–13]. Therefore, it is recommended that cancer patients’ physical activity and exercise beliefs and behaviors should be evaluated to provide appropriate guidance.

Physical activity level and exercise barriers of patients with cancer have been investigated in numerous studies. Majority of studies found poor physical activity level in patients with cancer during and after treatments [14]. A study assessing the physical activity levels of patients with breast cancer–related lymphedema found insufficient physical activity level in participants. The most prevalent barriers for engaging in physical activity were reported to be a lack of sufficient awareness and incorrect beliefs about exercise being potentially harmful [15]. While existing literature extensively covers the physical and psychological benefits of exercise in cancer patients [16–18], and some systematic reviews address patient barriers [19, 20], the specific interplay between positive exercise beliefs, perceived barriers, and their combined effect on physical activity level and social participation in patients undergoing active chemotherapy remains an under-researched area.

In a study, more than half of cancer patients had positive opinions and attitudes toward doing exercise, yet half of the patients reported exercise may be unsafe [21]. There is a need to investigate not only exercise barriers but also exercise beliefs in patients with cancer. On the other hand, physical activity levels have been found associated with numerous factors including symptoms and quality of life level in patients with cancer [14, 15]. To our knowledge, there is no study in the literature examining the effect of physical activity on individuals’ participation in social life. Therefore, the present study aimed to explore the physical activity levels and exercise beliefs of cancer patients undergoing outpatient chemotherapy, as well as their effects on participation and quality of life level.

## Material and method

The present study was conducted at Hacettepe University Oncology Hospital between April 2023 and December 2023. The inclusion criteria were being diagnosed with cancer and receiving outpatient chemotherapy, aged between 18 and 65 years, able to comply with assessments, and voluntarily signed the informed consent form. The study was approved by the Hacettepe University Non-Interventional Clinical Research Ethics Committee (Ethics Committee Approval Date: 18.04.2023, Approval Number: GO 23/343). Each participant was first informed about the study content, and their consent was obtained. Individuals with severe emotional problems, those who are unable to cooperate, or those unwilling to participate were excluded from the present study. The European Organization for Research and Treatment of Cancer Quality of Life Questionnaire (EORTC QLQ-C30) was used to evaluate the health-related quality of life level. The International Physical Activity Questionnaire (IPAQ) was used to measure physical activity levels, the Exercise Beliefs Questionnaire (EBQ) was used to determine exercise attitudes and beliefs, and the Reintegration to Normal Living Index (RNLI) was used to assess participation level.

Patient-reported outcome measures were used to collect data in the present study. Participants were asked to fill in the surveys; if needed, researchers asked the items to the patients. We collected data by face-to-face interviews when they were receiving chemotherapy. The following outcome measures were used.

### Demographic and information

Participants’ gender, age, height, weight, marital status, education level, and occupation were recorded. Medical information including diagnosis, time since diagnosis, surgical history, comorbidities, presence of lymphedema/neuropathy, chemotherapy and/or radiotherapy history, chronic diseases, medications used, mobility aids, and current treatment status was obtained from the hospital records.

### Performance status

Performance status (PS) is an estimate of a patient’s ability to perform daily living activities without assistance. The Eastern Cooperative Oncology Group (ECOG) performance scale was used to assess PS. The scores ranged from 0 to 4 points, where “0 points” represents a fully functional and asymptomatic patient and “4 points” represents a bedridden state [22].

### Quality of life level

The European Organization for Research and Treatment of Cancer Quality of Life Questionnaire Core 30 (EORTC QLQ-C30) was used to assess the health-related quality of life of participants. This questionnaire consists of three subcategories: general health, functional score, and symptom score. Each item is scored from 1 (not at all) to 4 (very much), with higher scores indicating better status for general health and functional scores, and worse status for symptom scores [23]. The Turkish version of the questionnaire has been validated [24].

### Physical activity level

The International Physical Activity Questionnaire (IPAQ) Short Form was used to assess physical activity level [25]. The questionnaire, which was adapted to Turkish by Ozturk et al. (2005), provides information on time spent walking, moderate-vigorous, and vigorous activities [26]. Time spent sitting is evaluated as a separate question. According to the total score, activity level is classified as inactive, minimally active, and active by calculating it in METs according to the duration, frequency, and intensity of the activity.

### Exercise beliefs

The Exercise Beliefs Questionnaire was originally developed by Doymaz et al. (2013). The scale was designed based on the Health Belief Model framework to evaluate individuals’ perceptions of exercise benefits and barriers. The questionnaire includes 33 items across three subscales: (A) Exercise Benefits, (B) Social Influence, and (C) Exercise Barriers, each rated on a 6-point Likert scale (1 = strongly disagree to 6 = strongly agree) [27]. Higher scores in parts A and B indicate stronger beliefs in exercise benefits and greater perceived support from others, whereas higher scores in part C reflect more perceived barriers. The EBQ demonstrated high internal consistency (Cronbach’s *α* = 0.83–0.91) and test–retest reliability (*r* = 0.87) [19].

### Participation level

Reintegration to Normal Living Index (RNLI) was used to measure level of participation. The RNLI assesses eight areas related to activities and daily living in the daily functions: mobility in the home, mobility in the community, ability to go out of town, self-care skills, daily and work activities, leisure activities, social roles, and family roles [28]. In the RNLI scale, 5-point Likert type was used as “Strongly agree,” “Agree,” “Undecided,” “Disagree,” and “Strongly disagree.” Total scores range from 11 to 55 points. A higher score indicates a better level of participation. The validity and reliability analyses of Turkish form were performed by Senem Demirdel in 2012 [29].

### Statistical analysis

All statistical analyses were performed using SPSS Version 23. Descriptive data were expressed as mean ± standard deviation (SD) or frequency (%). Data normality was tested with the Kolmogorov-Smirnov test. Since the data were not normally distributed, non-parametric tests were used. The Mann-Whitney *U* test was used to compare recorded data. The Spearman correlation coefficient (*r*) was used to examine the associations between physical activity level, exercise beliefs, social participation, and quality of life parameters. The strength of the correlations was interpreted using the following conventional criteria: 0.00–0.25 indicates a very weak to weak correlation, 0.25–0.50 indicates a weak to moderate correlation, 0.50–0.75 indicates a moderate to strong correlation, and > 0.75 indicates a strong to very strong correlation. A *p*-value of < 0.05 was considered statistically significant [30].

## Results

The present study included 101 individuals who were diagnosed with cancer and were receiving outpatient chemotherapy at Hacettepe University Oncology Hospital. Table 1 presents the demographic and medical characteristics of the participants. According to this, 58 (57.4%) of the participants were female, and 43 (42.6%) were male. Patients were middle-aged and according to the mean body mass index, they were classified as overweight (Table 1).

**Table 1:** Demographic and medical information of the participants

Recorded parameters	Mean ± SD Number (%)
Body mass index (BMI), kg/cm^2^	26.1 ± 4.8
Age, years	53.2 ± 12.8
Time since diagnosis, months	20.3 ± 35.3
Number of chemotherapy cycles	8.8 ± 7.4
Number of radiotherapy days	9.9 ± 15.6
Marital status	
Married	80 (79.2)
Single	20 (19.8)
Widowed	1 (1.0)
Education status	
Illiterate	2 (2.0)
Primary school	13 (12.9)
Secondary school	9 (8.9)
High school	30 (29.7)
University	41 (40.6)
Master’s/doctorate	6 (5.9)
Diagnosis	
Gastrointestinal system cancer	39 (38.6)
Breast cancer	29 (28.7)
Lung cancer	12 (11.5)
Lymphoma	6 (5.9)
Gynecological cancer	5 (5.0)
Pancreatic cancer	4 (4.0)
Liver cancer	2 (2.0)
Other	4 (4.0)
Surgical history	
Yes	70 (69.3)
No	31 (30.7)
Comorbidity	
Yes	59 (58.4)
No	42 (41.6)
Accompanying diseases	
Diabetes	18 (18.0)
Hypertension	14 (14.0)
Lung diseases	3 (3.0)
Thyroid disorders	2 (2.0)
Heart diseases	2 (2.0)
Other	5 (5.0)
Radiotherapy history	
Yes	70 (69.3)
No	31 (30.7)
Walking aid	
Yes	96 (95.0)
No	5 (5.0)
ECOG Performance Score	
0: Fully active	33 (32.7)
1: Restricted in physically strenuous activity	55 (54.5)
2: More than 50% of waking hours	13 (12.9)

*ECOG* Eastern Cooperative Oncology Group

During our evaluations, 98 (97.0%) of the patients were continuing chemotherapy, while 3 (2.9%) were receiving both chemotherapy and radiotherapy. The majority of participants were diagnosed with gastrointestinal system (38.6%) or breast cancer (28.7%). When the ECOG Performance Score was reviewed, most participants had a PS0 or PS1 score, which means that most patients could carry out their daily activities with manageable symptoms.

Table 2 presents the key findings regarding the participants’ physical activity levels, social participation, and quality of life parameters. According to the IPAQ, 55.4% of the participants were classified as minimally active, while 44.5% were inactive. Despite the low measured activity level, the participants reported a relatively high level of social participation, as indicated by the RNLI mean score (50.2 ± 4.1). Furthermore, the mean scores on the EORTC QLQ-C30 functional score (82.3 ± 14.1) and general health score (70.9 ± 22.6) were also high, suggesting that while their activity may be limited, their overall quality of life and engagement in life roles remained manageable (Table 2).

**Table 2:** Physical activity, exercise beliefs, quality of life, and participation level of the participants

Recorded parameters	Mean ± SD
EORTC QLQ-C30 function score	82.3 ± 14.1
EORTC QLQ-C30 symptom score	19.7 ± 14.4
EORTC QLQ-C30 general health score	70.9 ± 22.6
RNLI total score	50.2 ± 4.1
IPAQ-SF, total MET-min/week	961.8 ± 1208.0
IPAQ-Sitting, hours	7.4 ± 2.6
IPAQ activity classification, *n* (%)	
Inactive	45 (44.5)
Minimally active	56 (55.4)
Exercise Beliefs Total Score	
Inactive	61.4 ± 13.9
Minimally active	64.7 ± 13.3
*p*-value	0.121
Exercise Barriers Total Score	
Inactive	33.4 ± 8.2
Minimally active	30.0 ± 9.2
*p*-value	0.021

*SD* standard deviation, *EORTC QLQ-C30* European Organization for the Research and Treatment of Cancer Quality of Life Questionnaire Core 30, *RNLI* Reintegration to Normal Living Index, *IPAQ-SF* International Physical Activity Questionnaire-Short Form, *MET* metabolic equivalent

Table 3 presents the participants’ exercise beliefs and perceived barriers. Regarding exercise benefits, patients reported consistently high positive beliefs, with the strongest agreement being that exercise makes them feel better (5.4 ± 1.1), reduces stress (5.3 ± 1.2), and positively affects their mood (5.3 ± 1.3). Conversely, the major perceived exercise barriers were reported with notably high scores. The most significant barriers included fatigue/lack of energy (4.5 ± 1.7), bad weather conditions (4.0 ± 2.1), lack of motivation (3.9 ± 1.9), and closely followed by health problems (3.8 ± 1.9) (Table 3).

**Table 3:** Participants’ exercise beliefs and barriers

	Mean ± SD
Beliefs about exercise benefits	
1. Exercise positively affects my mood	5.3 ± 1.3
2. Exercise helps me get less tired	4.1 ± 1.8
3. Exercise protects me from illnesses	4.6 ± 1.6
4. Exercise makes me feel better	5.4 ± 1.1
5. Exercise improves my physical appearance	5.0 ± 1.5
6. Exercise reduces my stress and helps me relax	5.3 ± 1.2
7. Exercise prevents weight gain	4.7 ± 1.6
8. Exercise makes my daily life easier	4.7 ± 1.3
9. Exercise is beneficial for my heart and vascular health	4.9 ± 1.4
10. Exercise is protective against diabetes	4.7 ± 1.5
11. Exercise strengthens my immune system	4.9 ± 1.4
12. Exercise helps me lose weight	4.6 ± 1.7
13. Exercise is protective against chronic diseases	4.9 ± 1.4
Total score	63.2 ± 13.6
Exercise barriers	
1. I have health problems	3.8 ± 1.9
2. I’m tired, I have no energy	4.5 ± 1.7
3. I can’t find time	2.1 ± 1.6
4. I’m overweight	1.8 ± 1.3
5. I’m afraid of injuring myself	2.2 ± 1.9
6. Bad weather conditions	4.0 ± 2.1
7. I’m not willing or I feel lazy	3.9 ± 1.9
8. I don’t think it’s necessary	2.0 ± 1.7
9. I can’t afford it	1.6 ± 1.3
10. I don’t like exercising	2.0 ± 1.5
11. I don’t have a workout partner	1.80 ± 1.5
12. There is no suitable environment for exercise	1.5 ± 1.2
Total score	31.5 ± 8.9

Table 4 provides data on the influence of other people on participants’ exercise habits. The results showed strong positive support from the participants’ social circle. Specifically, healthcare workers (93.1%), relatives (89.1%), spouses/partners, parents, and children (82.2%) were reported as the primary source of positive support for physical activity (Table 4).

**Table 4:** Opinions on how individuals affect participants

How …… affect you to do exercise?	Positive *N* (%)	Negative *N* (%)
1. Spouse, partner	83 (82.2)	18 (17.8)
2. Parents	83 (82.2)	18 (17.8)
3. Children	83 (82.2)	18 (17.8)
4. Relatives	90 (89.1)	11 (10.9)
5. Friends	91 (90.1)	10 (9.9)
6. Healthcare workers	94 (93.1)	7 (6.9)
7. Sports experts	97 (96.0)	4 (4.0)
8. Other people	98 (97.0)	3 (3.0)

The correlation analysis between physical activity level and the measured parameters is shown in Table 5. Correlation analysis revealed significant associations between physical activity levels, exercise beliefs, social participation, and quality of life parameters. Physical activity scores showed a moderate positive correlation with social participation (*r* = 0.453,* p* < 0.001). Furthermore, physical activity was positively correlated with EORTC QLQ-C30 Functional Scale scores (*r* = 0.331,* p* = 0.001) and EORTC QLQ-C30 Global Health scores (*r* = 0.247,* p* = 0.013). A weak but significant negative correlation was observed between physical activity and EORTC QLQ-C30 Symptom Scale scores, indicating that higher activity was associated with fewer symptoms (*r* = −0.259,* p* = 0.009). Crucially, negative exercise beliefs and barriers demonstrated significant negative moderate correlations with RNLI total score (*r* = −0.388, *p* < 0.001), EORTC QLQ-C30 Functional Scale score (*r* = −0.362, *p* < 0.001), and EORTC QLQ-C30 Global Health scores (*r* = −0.240, *p* = 0.016), while positively correlating with EORTC QLQ-C30 Symptom Scale scores (*r* = 0.309, *p* = 0.002). Regarding sedentary behavior, higher sitting time was significantly associated with lower positive exercise beliefs (*r* = −0.279, *p* = 0.005) and higher perceived exercise barriers (*r* = 0.215, *p* = 0.030) (Table 5).

**Table 5:** Correlations between physical activity levels, exercise beliefs, social participation, and quality of life

Correlation analysis	IPAQ Total Score	EBQ-exercise benefits	EBQ-exercise barriers
Patient-reported outcomes and quality of life			
EORTC QLQ-C30 Function Score	0.331*	−	−0.362**
EORTC QLQ-C30 Symptom Score	−0.259*	−	0.309*
EORTC QLQ-C30 General Health Score	0.247*	−	−0.273*
RNLI Total Score	0.453**	−	−0.388**
Physical activity subscales			
IPAQ Total Score	−	0.308*	−0.273*
Sitting time	−	−0.279*	0.215*

*EORTC QLQ-C30 * European Organization for the Research and Treatment of Cancer Quality of Life Questionnaire Core 30, *RNLI* Reintegration to Normal Living Index, *IPAQ* International Physical Activity Questionnaire, *EBQ* Exercise Beliefs Questionnaire, The Spearman correlation coefficient test, *p < 0.05, **p < 0.001

## Discussion

The present study examined the physical activity levels and exercise beliefs of patients with cancer receiving outpatient chemotherapy and investigated their associations with exercise barriers, social participation, and quality of life level. The results showed that nearly half of the patients were inactive, and the rest were minimally active. When their beliefs about exercise were examined, most participants reported having positive beliefs, and were influenced positively by those relatives. The primary barriers to physical activity were health problems, fatigue, and lack of motivation to do exercise. A significant finding was that patients with higher physical activity level reported significantly higher quality of life, participation, and lower exercise barrier. Moreover, lower negative exercise beliefs were associated with better quality of life and social participation.

The present study focused on patients receiving outpatient chemotherapy, with the most prevalent diagnoses being gastrointestinal system cancer (38.6%) and breast cancer (28.7%). The clinical characteristics of our sample, particularly their functional status, are crucial for interpreting the activity data. An examination of the ECOG Performance Status revealed that the majority of participants (54.1% with PS1) maintained a relatively high level of function, meaning they were able to carry out daily activities but were restricted only in strenuous physical activity. This distribution is consistent with the previous research conducted in similar outpatient chemotherapy settings [31], suggesting that our cohort represents a clinically stable population.

Several reviews and meta-analyses provide strong evidence that physical activity and exercise are safe and acceptable before, during, and after cancer treatment in all types of cancer [32], including advanced-stage cancer patients receiving palliative treatment [33]. Our primary finding reveals a persistent challenge in exercise oncology: nearly all participants were classified as inactive (44.5%) or minimally active (55.4%). This is observed despite strong evidence that physical activity and exercise are safe and highly beneficial before, during, and after cancer treatment in all types of cancer [32, 33]. A meta-analysis has indicated that exercise training is an effective non-pharmacological treatment method that contributes to symptom management by reducing the adverse effects of treatment, including inflammation in cancer survivors [33]. In a similar study conducted by Yıldız Kabak et al., nearly 50% of patients with breast cancer–related lymphedema were found to be inactive and 50% were minimally active [13]. This demonstrates that while the physical capability may exist (as shown by their ECOG status), the transition to regular physical activity is a widespread issue in the outpatient setting. If exercise beliefs and barriers are explored, the reasons for this low physical activity level may be determined. In this way, effective strategies such as lifestyle modifications and behavioral strategies could be planned.

According to the results of the RNLI, the mean score of the participants indicates that the individuals have largely re-entered normal life and low social participation restrictions. However, a closer examination of the scale items revealed a key area of difficulty: most of the participants disagreed with the option “I can travel out of town when necessary.” While the RNLI is a reliable and valid measure of community integration for those living with chronic health conditions, its use in cancer populations has been rare. This limited our ability to directly compare our results with other cancer-specific studies, although similar findings were also found in the other populations. Bodechtel et al. reported a mean RNLI score of 54.98 points in sepsis survivors [34], and another study in extremity sarcoma patients found low-level participation restrictions [35]. Although our results are similar to those of different chronic diseases, there is a need for scales that specifically assess participation limitations in cancer patients. Since the performance of activities and participation in life roles are often the main goals of rehabilitation, measuring the activity and participation subdomains in cancer patients may provide useful information about the necessity and effectiveness of oncological rehabilitation.

The most significant finding of our study is the discrepancy between exercise motivation and action. Participants reported consistently high scores on positive exercise beliefs, agreeing that exercise makes them feel better, reduces stress, and positively affects their mood. This positive attitude, which aligns with previous research [36], suggests that primary barriers to activity may not lack of knowledge or belief in the benefits of exercise. Instead, the data strongly points toward the overwhelming impact of perceived barriers. The most significant obstacles identified were the physical and psychological burdens of chemotherapy: fatigue/lack of energy, health problems, and lack of motivation. This confirms the priority of treatment side effects as the main impediment to exercise adherence, consistent with a systematic review by Clifford et al. [19] and a previous study by Romero et al. [37] highlighted major barriers as fatigue, pain, and difficulty in motivation.

Crucially, our analysis showed that inactive patients experienced significantly higher barriers compared to minimally active patients. This outcome is consistent with the previous findings [19] and it may suggest that while promoting positive beliefs is foundational, a focus on actively dismantling the physical and psychological barriers may be the crucial intervention point for bridging the intention-to-behavior gap and increasing patients’ self-efficacy.

A significant finding of the present study was that negative exercise beliefs and barriers played a more decisive role on clinical and functional outcomes than positive beliefs. Our correlation analysis demonstrated that higher negative beliefs and barriers were significantly associated with increased symptom scores, lower functional outcomes, and poorer social participation. In the literature, Clifford et al. [19] similarly emphasized that perceived barrier, rather than a lack of knowledge about exercise benefits, acts as a primary limiting factor for physical activity participation in cancer survivors. Additionally, Finch and Benham [20] reported that negative attitudes driven by treatment side effects directly translate into lower quality of life. Our study extends these findings by demonstrating that negative exercise beliefs clearly hinder the patient’s reintegration into normal living and social roles. Therefore, oncological rehabilitation programs should focus on identifying and reducing specific negative beliefs and perceived barriers to improve physical activity and participation levels. The present study showed that the physical activity levels of individuals receiving chemotherapy are significantly related to quality of life and participation in social life. Low physical activity level may negatively affect the reintegration of individuals into their life roles. Studies on the relationship between physical activity and quality of life have generally been conducted on individuals with breast cancer and found significant association between physical activity level and quality of life level [38–40].

To our knowledge, there are only a limited number of studies in literature that directly examine the relationship between physical activity and social participation level in individuals with cancer. A previous study showed that individuals with high levels of physical activity participated in activities of daily life more frequently than those who were less physically active [41]. In a study conducted by Demirdel et al. on the relationship between the process of re-engagement in normal life after amputation and quality of life and functional level, it was shown that participation in normal life after amputation was associated with functional level [29]. Similar with these findings, we showed that as physical activity level increased, patients’ participation into daily life increased among cancer patients receiving chemotherapy.

The relatively small number of participants included in the study limited the generalizability of the results. A larger sample size could better represent different types and stages of cancer, thereby increasing the external validity of the findings. Furthermore, the fact that the participants were mostly recruited from a specific geographic region or health center reduced the diversity of the results. Methodologically, the use of only last week’s data to determine physical activity levels constitutes a limitation, as it may overlook temporal fluctuations in activity patterns. Additionally, the reliance on self-report questionnaires means the data are based on the participants’ own perceptions and statements, which may limit the objectivity of the responses. Therefore, future studies are needed to employ objective measurement tools to evaluate physical activity level, such as accelerometers, over a more extended period, including diverse cohorts to fully understand the long-term impact of these factors. In addition, since psychological factors such as depression, anxiety, or stress that may affect the relationship between physical activity and quality of life, it should be further investigated in the future.

The subjective nature of the questionnaire data and the potential confounding effect of psychological factors (such as depression, anxiety, or stress) highlight the necessity of adopting a holistic intervention framework. Future programs should address the psychological and motivational barriers (e.g., unwillingness to do exercise) that prevent participants from translating their positive exercise beliefs into action. Effective multi-dimensional interventions including physical activity, motivational interviewing strategies, cognitive-behavioral therapy, or group support should integrate components aimed at enhancing self-efficacy and managing psychological distress. Furthermore, considering the participants’ stated difficulty with travelling, intervention programs may increasingly feature local, accessible, or home-based options to minimize environmental barriers to participation in physical activity programs.

## Conclusion

Our results revealed that while patients undergoing chemotherapy hold positive exercise beliefs, they exhibited insufficient physical activity levels. Crucially, higher physical activity and lower negative exercise beliefs were associated with better social participation and quality of life. This finding suggests that simply promoting the benefits of exercise may be inadequate to promote physical activity behavior. Effective physical activity interventions may shift its focus to actively reducing the primary, modifiable barriers of fatigue, lack of motivation, and health concerns through targeted personalized interventions. Future programs should integrate strategies—such as motivational interviewing and accessible, home-based options—to enhance patient self-efficacy, manage psychological distress, and translate positive beliefs into sustained behavior, thereby promoting greater re-engagement in normal living.

## Data Availability

No datasets were generated or analysed during the current study.

## References

[1] Ülger E, Alacacıoğlu A, Gülseren AŞ, Zencir G, Demir L, Tarhan MO (2014) Kanserde Psikososyal Sorunlar Ve Psikososyal Onkolojinin Önemi,. Dokuz Eylul Univ Tip Fak Derg 28(2):85–92. 10.18614/deutfd.88761

[2] Bevans MF, Sternberg EM (2012) Caregiving burden, stress, and health effects among family caregivers of adult cancer patients. JAMA 307(4):398–403. 10.1001/jama.2012.29 22274687 10.1001/jama.2012.29 PMC3304539

[3] Publishing Commission of General Directorate of Public Health, Türkiye Oncology Cancer Control Programme (2021) Ankara. https://hsgm.saglik.gov.tr/depo/birimler/kanser-db/Dokumanlar/Raporlar/Turkey_NCCP_18_April_2022.pdf. Accessed 18 April 2022

[4] Keser İ, Özdemir K, Ertürk B, Haspolat M, Duman T, Esmer M (2017) The analysis of the provided services to cancer patients at oncological rehabilitation unit. Gazi J of Health Sciences 2(1):18–27

[5] Yılmaz RC, Kocamaz D, Ayhan Fidancıoğlu N, Yıldırım M (2022) Exercise-based oncological rehabilitation in cancer patients. J Med Top Updates 1(2):85–92. 10.5281/zenodo.6989657

[6] Patel AV, Friedenreich CM, Moore SC, Hayes SC, Silver JK, Campbell KL et al (2019) American College of Sports Medicine roundtable report on physical activity, sedentary behavior, and cancer prevention and control. Med Sci Sports Exerc 51(11):2391–2402. 10.1249/MSS.0000000000002117 31626056 10.1249/MSS.0000000000002117 PMC6814265

[7] De Raaf C, De Klerk C, Van Der Rijt C (2013) Elucidating the behavior of physical fatigue and mental fatigue in cancer patients: a review of the literature. Psycho-Oncol 22(9):1919–1929. 10.1002/pon.3225 10.1002/pon.3225 23147803

[8] Misiąg W, Piszczyk A, Szymańska-Chabowska A, Chabowski M (2022) Physical activity and cancer care-a review. Cancers 14(17):4154. 10.3390/cancers14174154 36077690 10.3390/cancers14174154 PMC9454950

[9] Jette AM (2006) Toward a common language for function, disability, and health. Phys Ther 86(5):726–734. 10.1093/ptj/86.5.726 16649895

[10] Sperlich S, Noeres D, Holthausen-Markou S, Park-Simon TW, Sahiti E, Geyer S (2024) Social participation of women with breast cancer compared to the general population 5 years after primary surgery-what role do medical data and cancer-related complaints play. Support Care Cancer 32(8):566. 10.1007/s00520-024-08695-w 39093481 10.1007/s00520-024-08695-w PMC11297071

[11] Ballard-Barbash R, Friedenreich CM, Courneya KS, Siddiqi SM, McTiernan A, Alfano CM (2012) Physical activity, biomarkers, and disease outcomes in cancer survivors: a systematic review. J Natl Cancer Inst 104(11):815–840. 10.1093/jnci/djs207 22570317 10.1093/jnci/djs207 PMC3465697

[12] Rock CL, Doyle C, Demark-Wahnefried W et al (2012) Nutrition and physical activity guidelines for cancer survivors. CA Cancer J Clin 62(4):243–274. 10.3322/caac.21142 22539238 10.3322/caac.21142

[13] Yildiz Kabak V, Gursen C, Aytar A et al (2021) Physical activity level, exercise behavior, barriers, and preferences of patients with breast cancer-related lymphedema. Support Care Cancer 29:3593–3602. 10.1007/s00520-021-06051-7 33170403 10.1007/s00520-020-05858-3

[14] Blaney JM, Lowe-Strong A, Rankin-Watt J, Campbell A, Gracey JH (2013) Cancer survivors’ exercise barriers, facilitators and preferences in the context of fatigue, quality of life and physical activity participation: a questionnaire-survey. Psychooncology 22(1):186–194. 10.1002/pon.2072 23296635 10.1002/pon.2072

[15] Thraen-Borowski KM, Trentham-Dietz A, Edwards DF, Koltyn KF, Colbert LH (2013) Dose–response relationships between physical activity, social participation, and health-related quality of life in colorectal cancer survivors. J Cancer Surviv 7(3):369–378. 10.1007/s11764-013-0277-7 23546822 10.1007/s11764-013-0277-7 PMC3737238

[16] Albini A, La Vecchia C, Magnoni F, Garrone O, Morelli D, Janssens JP et al (2025) Physical activity and exercise health benefits: cancer prevention, interception, and survival. Eur J Cancer Prev 34(1):24–39. 10.1097/cej.0000000000000898 38920329 10.1097/CEJ.0000000000000898

[17] Sánchez-González JL, Fernández-Rodríguez EJ, Méndez-Sánchez R, Polo-Ferrero L, Puente-González AS, de Ramón C et al (2025) Effects of a strength physical exercise program in chronic lymphocytic leukemia patients on quality of life, mental health, and frailty: a randomized controlled trial study protocol. Front Sports Act Living 7:1534861. 10.3389/fspor.2025.1534861 40129522 10.3389/fspor.2025.1534861 PMC11931114

[18] Li A, Zheng X, Liu D, Huang R, Ge H, Cheng L et al (2025) Physical activity and depression in breast cancer patients: mechanisms and therapeutic potential. Curr Oncol 32(2):77. 10.3390/curroncol32020077 39996878 10.3390/curroncol32020077 PMC11854877

[19] Clifford BK, Mizrahi D, Sandler CX, Barry BK, Simar D, Wakefield CE, Goldstein D (2018) Barriers and facilitators of exercise experienced by cancer survivors: a mixed methods systematic review. Support Care Cancer 26(3):685–700. 10.1007/s00520-017-3964-5 29185105 10.1007/s00520-017-3964-5

[20] Finch A, Benham A (2024) Patient attitudes and experiences towards exercise during oncological treatment. A qualitative systematic review. Support Care Cancer 32:509. 10.1007/s00520-024-08649-2 10.1007/s00520-024-08649-2 PMC11239782 38992238

[21] Foucaut AM, Berthouze SE, Touillaud M et al (2015) Deterioration of physical activity level and metabolic risk factors after early-stage breast cancer diagnosis. Cancer Nurs 38(4):1–9. 10.1097/NCC.0000000000000187 25207592 10.1097/NCC.0000000000000187

[22] Çeltek NY, Okan İ (2016) The evaluation of palliative care patient and used scales. J Clin Med Fam Med 8(3):6–10

[23] Aaronson NK, Ahmedzai S, Bergman B et al (1993) The European Organization for Research and Treatment of Cancer QLQ-C30: a quality-of-life instrument for use in international clinical trials in oncology. J Natl Cancer Inst 85(5):365–376. 10.1093/jnci/85.5.365 8433390 10.1093/jnci/85.5.365

[24] Guzelant A, Goksel T, Ozkok S et al (2004) The European Organization for Research and Treatment of Cancer QLQ-C30: an examination into the cultural validity and reliability of the Turkish version of the EORTC QLQ-C30. Eur J Cancer Care 13(2):135–144. 10.1111/j.1365-2354.2004.00450.x 10.1111/j.1365-2354.2003.00435.x 15115469

[25] Craig CL, Marshall AL, Sjöström M et al (2003) International physical activity questionnaire: 12-country reliability and validity. Med Sci Sports Exerc 35(8):1381–1395. 10.1249/01.MSS.0000078924.61453.FB 12900694 10.1249/01.MSS.0000078924.61453.FB

[26] Öztürk M (2005) A research on reliability and validity of international physical activity questionnaire and determination of physical activity level in university students. Master Thesis, Hacettepe University Health Sciences Institute, Ankara

[27] Doymaz F (2013) Examination of the impact of exercise beliefs on exercise behavioral changes in healthy women. Doctoral Thesis, Hacettepe University Health Sciences Institute, Ankara

[28] Nissen SJ, Newman WP (1992) Factors influencing reintegration to normal living after amputation. Arch Phys Med Rehabil 73(6):548–551. 10.5555/jrm.v56.18670 1622303

[29] Demirdel S, Bayramlar K (2016) Investigation of the relationship of reintegration process to normal living after amputation with quality of life and functional level. Turk J Physiother Rehabil 25(3):93–99. 10.1177/1539449220905794

[30] Portney LG, Watkins MP (2009) Foundations of clinical research: applications to practice, 3rd edn. Pearson Education, Upper Saddle River, NJ

[31] Stout NL, Baima J, Swisher AK et al (2017) A systematic review of exercise systematic reviews in the cancer literature (2005-2017). PM R 9(9S2):347–384. 10.1016/j.pmrj.2017.07.074 10.1016/j.pmrj.2017.07.074 PMC5679711 28942909

[32] Oldervoll LM, Loge JH, Lydersen S et al (2011) Physical exercise for cancer patients with advanced disease: a randomized controlled trial. Oncologist 16(11):1649–1657. 10.1634/theoncologist.2011-0133 21948693 10.1634/theoncologist.2011-0133 PMC3233301

[33] Khosravi N, Stoner L, Farajivafa V, Hanson ED (2019) Exercise training, circulating cytokine levels and immune function in cancer survivors: a meta-analysis. Brain Behav Immun 81:92–104. 10.1016/j.bbi.2019.08.187 31454519 10.1016/j.bbi.2019.08.187

[34] Bodechtel U, Koch T, Heubner L, Spieth P, Rößler I, Mehrholz J (2024) Health-related quality of life and participation after inpatient rehabilitation of sepsis survivors with severe sequelae: a cohort study. J Rehabil Med 56:jrm18670. 10.2340/jrm.v56.18670 38956963 10.2340/jrm.v56.18670 PMC11247512

[35] Blight TJ, Choong PFM (2021) The need for improved patient-reported outcome measures in patients with extremity sarcoma: a narrative review. ANZ J Surg 91(10):2021–2025. 10.1111/ans.17028 34227237 10.1111/ans.17028

[36] Caperchione CM, Stolp S, Phillips JL et al (2022) Cancer survivors’ exercise beliefs, knowledge, and behaviors: an Australian National Survey. Asia Pac J Clin Oncol 18(6):625–633. 10.1111/ajco.13729 35098655 10.1111/ajco.13729

[37] Romero-Elías M, Beltrán-Carrillo VJ, González-Cutre D, Jiménez-Loaisa A (2020) Barriers to physical activity participation in colorectal cancer patients during chemotherapy treatment: a qualitative study. Eur J Oncol Nurs 46:101769. 10.1016/j.ejon.2020.101769 32506009 10.1016/j.ejon.2020.101769

[38] De Vrieze T, Gebruers N, Nevelsteen I et al (2020) Physical activity level and age contribute to functioning problems in patients with breast cancer-related lymphedema: a multicentre cross-sectional study. Support Care Cancer 28:5717–5731. 10.1007/s00520-020-05375-3 32193692 10.1007/s00520-020-05375-3

[39] Sun M, Liu C, Lu Y, Zhu F, Li H, Lu Q (2023) Effects of physical activity on quality of life, anxiety, and depression in breast cancer survivors: a systematic review and meta-analysis. Asian Nurs Res (Korean Soc Nurs Sci) 17(5):276–285. 10.1016/j.anr.2023.11.001 37944798 10.1016/j.anr.2023.11.001

[40] Solberg Nes L, Liu H, Patten CA et al (2012) Physical activity level and quality of life in long-term lung cancer survivors. Lung Cancer 77(3):611–616. 10.1016/j.lungcan.2012.05.096 22681871 10.1016/j.lungcan.2012.05.096 PMC3882512

[41] Crawford A, Hollingsworth HH, Morgan K, Gray DB (2008) People with mobility impairments: physical activity and quality of participation. Disabil Health J 1(1):7–13. 10.1016/j.dhjo.2007.11.004 21122706 10.1016/j.dhjo.2007.11.004

